# Immunometabolic programming of macrophages and tumor-associated macrophages: metabolic pathways shaping polarization, immune regulation, and disease outcomes

**DOI:** 10.3389/fimmu.2026.1803827

**Published:** 2026-09-24

**Authors:** Jiawei He, Bowen Wang, Enze Lei, Ningfeng Mao

**Affiliations:** 1 College of Traditional Chinese Medicine, Hubei University of Chinese Medicine, Wuhan, China; 2 Hubei Shizhen Laboratory, Wuhan, China; 3 Department of Traditional Chinese Medicine, Renmin Hospital of Wuhan University, Wuhan, China; 4 Department of Pediatrics, Hubei Provincial Hospital of Traditional Chinese Medicine, Affiliated Hospital of Hubei University of Chinese Medicine, Wuhan, China; 5 Wuhan Hospital of Traditional Chinese Medicine, Wuhan, China

**Keywords:** ferroptosis, glycolysis and oxidative phosphorylation, lipid metabolism, macrophage immunometabolism, metabolic reprogramming, tumor-associated macrophages

## Abstract

Macrophages adapt to hanging tissue environments by rewiring cellular metabolism, and these metabolic programs actively instruct immune functions rather than merely supplying energy. Across health and disease, shifts in glycolysis, mitochondrial oxidative phosphorylation, lipid handling, and amino acid utilization shape macrophage polarization, inflammatory mediator production, antigen presentation, and interactions with lymphocytes. In tumors, these principles are illustrated by tumor-associated macrophages (TAMs), which are reprogrammed by hypoxia, nutrient competition, and tumor-derived metabolites to adopt phenotypes that frequently support immune evasion, angiogenesis, extracellular matrix remodeling, and therapy resistance. This review integrates core macrophage immunometabolic pathways with TAM biology, emphasizing how lactate accumulation, altered lipid mediators, cholesterol remodeling, and amino acid dependencies reshape macrophage states and suppress antitumor immunity through checkpoint signaling and metabolic deprivation. We also discuss how immunometabolism intersects with regulated cell death, highlighting ferroptosis as an emerging mechanism linking iron metabolism, lipid peroxidation, redox control, and immune outcomes. Finally, we summarize therapeutic opportunities targeting metabolic nodes in tumor cells and macrophages, including modulation of lactate transport, fatty acid oxidation and synthesis, arginine/tryptophan pathways, and PI3Kγ-linked programs, and consider how interspecies differences between murine and human macrophages influence translational interpretation. Together, these insights position macrophage immunometabolism as a tractable framework for designing interventions that reshape immune responses in cancer and other macrophage-driven diseases.

## 1 Introduction

Macrophages are central regulators of innate and adaptive immunity, displaying remarkable functional plasticity that enables them to respond dynamically to diverse environmental cues (1). Traditionally classified as pro-inflammatory or anti-inflammatory, macrophages orchestrate immune defense, tissue repair, and homeostasis across physiological and pathological contexts (2). Increasingly, metabolism has emerged as a decisive determinant of macrophage identity and function, fueling the rapidly expanding field of macrophage immunometabolism (3). Rather than acting as passive suppliers of energy, metabolic pathways actively shape macrophage fate and immune behavior (4). Glycolysis, oxidative phosphorylation (OXPHOS), lipid metabolism, and amino acid utilization are now recognized as key regulators of macrophage polarization, cytokine production, antimicrobial activity, and interactions with other immune cells (5). Pro-inflammatory macrophages typically engage aerobic glycolysis and truncated tricarboxylic acid (TCA) cycle activity, supporting rapid effector responses (6). In contrast, anti-inflammatory macrophages rely on intact mitochondrial respiration, fatty acid oxidation, and anabolic lipid pathways (7). These metabolic processes are highly adaptable and can be reshaped by microenvironmental factors, including hypoxia, nutrient competition, microbial products, and tissue-derived metabolites.

Although macrophage immune metabolism is closely linked to infection, autoimmunity, and metabolic diseases, this review specifically emphasizes tumor-associated macrophages to illustrate how metabolic reprogramming shapes immunosuppression, tissue remodeling, and therapeutic responses. Insights from macrophage biology provide transferable frameworks for other pathological processes driven by macrophages.

The immune metabolism of macrophages has profound effects in a wide range of diseases. In infections, metabolic reconfiguration determines the pathogen’s antibacterial activity and persistence (8). In chronic inflammation and autoimmune diseases, dysregulated macrophage metabolism sustains pathogenic immune activation. In cancer and other metabolic diseases, the tissue microenvironment imposes metabolic limitations, prompting macrophages to shift to an immunosuppressive phenotype, thereby affecting disease progression and therapeutic responses (9). It is important that the metabolic state of macrophages also regulates cell death pathways, including ferroptosis, which, in turn, further regulates immune outcomes (10). Despite major advances, key challenges remain. The classical M1/M2 paradigm cannot fully capture macrophage metabolic states, as single-cell and spatial analyses reveal extensive heterogeneity shaped by tissue context and metabolic inputs (11). Moreover, significant interspecies differences in macrophage metabolism exist between murine and human systems, complicating translational interpretation. Addressing these gaps is essential for advancing macrophage-targeted therapies **Figure 1**.

**Figure 1:**
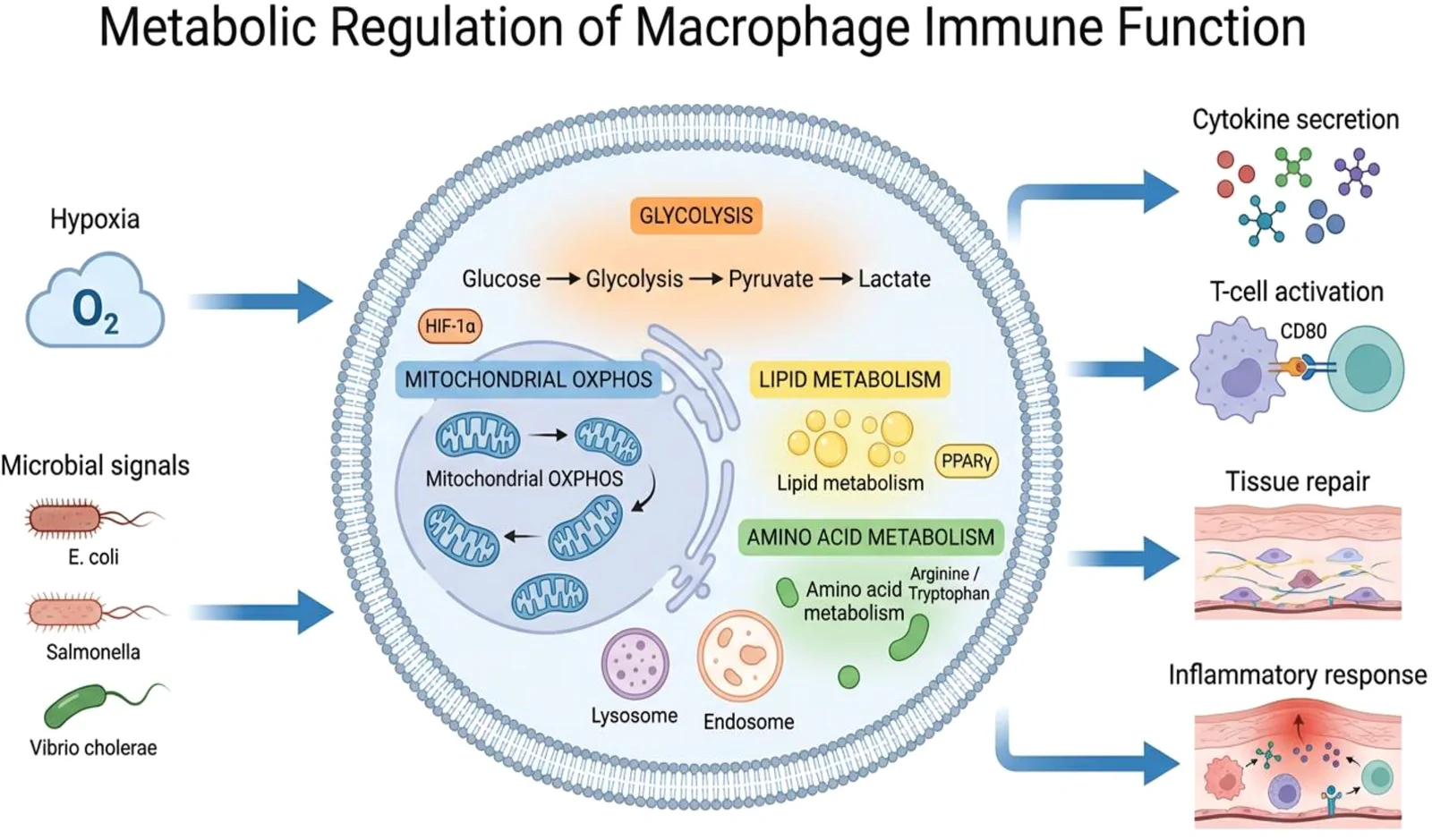
Overview of macrophage immunometabolism. Key metabolic pathways, including glycolysis, oxidative phosphorylation, lipid metabolism, and amino acid metabolism, regulate macrophage activation, polarization, and immune functions.

In this review, we provide an integrated overview of macrophage immunometabolism, linking core metabolic pathways to immune regulation, disease mechanisms, and therapeutic opportunities. Unlike previous reviews that primarily focus on macrophage polarization or individual metabolic pathways, this review integrates macrophage immunometabolism with tumor-associated macrophage heterogeneity, metabolic crosstalk within the tumor microenvironment, ferroptosis-related mechanisms, and translational differences between murine and human macrophages. By bridging metabolic and immune pathways, we aim to offer a unified framework for understanding how metabolic adaptations govern macrophage function in health and disease.

## 2 Core metabolic pathways governing macrophage immunometabolism

Macrophage function is fundamentally constrained by intracellular metabolic pathways that integrate environmental signals with immune effector programs (12). Under different tissue and disease conditions, glycolysis, mitochondrial oxidative phosphorylation (OXPHOS), lipid metabolism, and amino acid consumption jointly constitute the basic metabolic axis that controls macrophage activation, polarization, and immune activity (13). Proinflammatory macrophages usually exhibit a metabolic profile dominated by glycolysis, characterized by increased glucose uptake, accelerated pentose phosphate (PPP) pathway activity, and disruption of the tricarboxylic acid (TCA) cycle (14). This metabolic arrangement can rapidly generate ATP, produce reactive oxygen species (ROS), and synthesize inflammatory mediators necessary for combating bacteria and tumors. By stabilizing hypoxia-inducible factor-1α (HIF-1α), the accumulation of metabolic intermediates such as succinate increases inflammatory signals.

In contrast, anti-inflammatory and tissue repair-type macrophages rely on complete mitochondrial metabolism, including oxidative phosphorylation and fatty acid oxidation (FAO) (15). These pathways can maintain long-term survival, biosynthetic potential, and the generation of anti-inflammatory cytokines. Alternative activation processes and immune regulatory activities are strengthened through lipid uptake, storage, and signal transduction via nuclear receptors (such as peroxisome proliferator-activated receptor-γ (PPARγ)) (16). Another layer of regulation comes from amino acid metabolism. Arginine, glutamine, and tryptophan metabolism not only provides biosynthetic precursors but also shapes macrophage phenotypes and interacts with other immune cells, regulating metabolites (17). Collectively, these metabolic pathways establish a dynamic and adaptable framework that enables macrophages to respond to diverse immunological challenges **Figure 2**. Beyond supplying bioenergetic support, these metabolic pathways function as signaling hubs that regulate macrophage transcriptional programs. Metabolic intermediates such as succinate, citrate, and lactate can directly modulate inflammatory signaling pathways and epigenetic regulation, thereby linking metabolic flux to macrophage immune phenotypes. It is important to note that much of our mechanistic understanding of macrophage immunometabolism derives from murine experimental systems, and several metabolic features differ between mouse and human macrophages. For example, inducible nitric oxide synthase (iNOS)–dependent arginine metabolism is a dominant feature of inflammatory macrophages in mice. In contrast, human macrophages often display lower nitric oxide production and rely more heavily on alternative metabolic and redox regulatory pathways (18). These interspecies differences should be considered when translating metabolic insights from experimental models to human disease.

**Figure 2:**
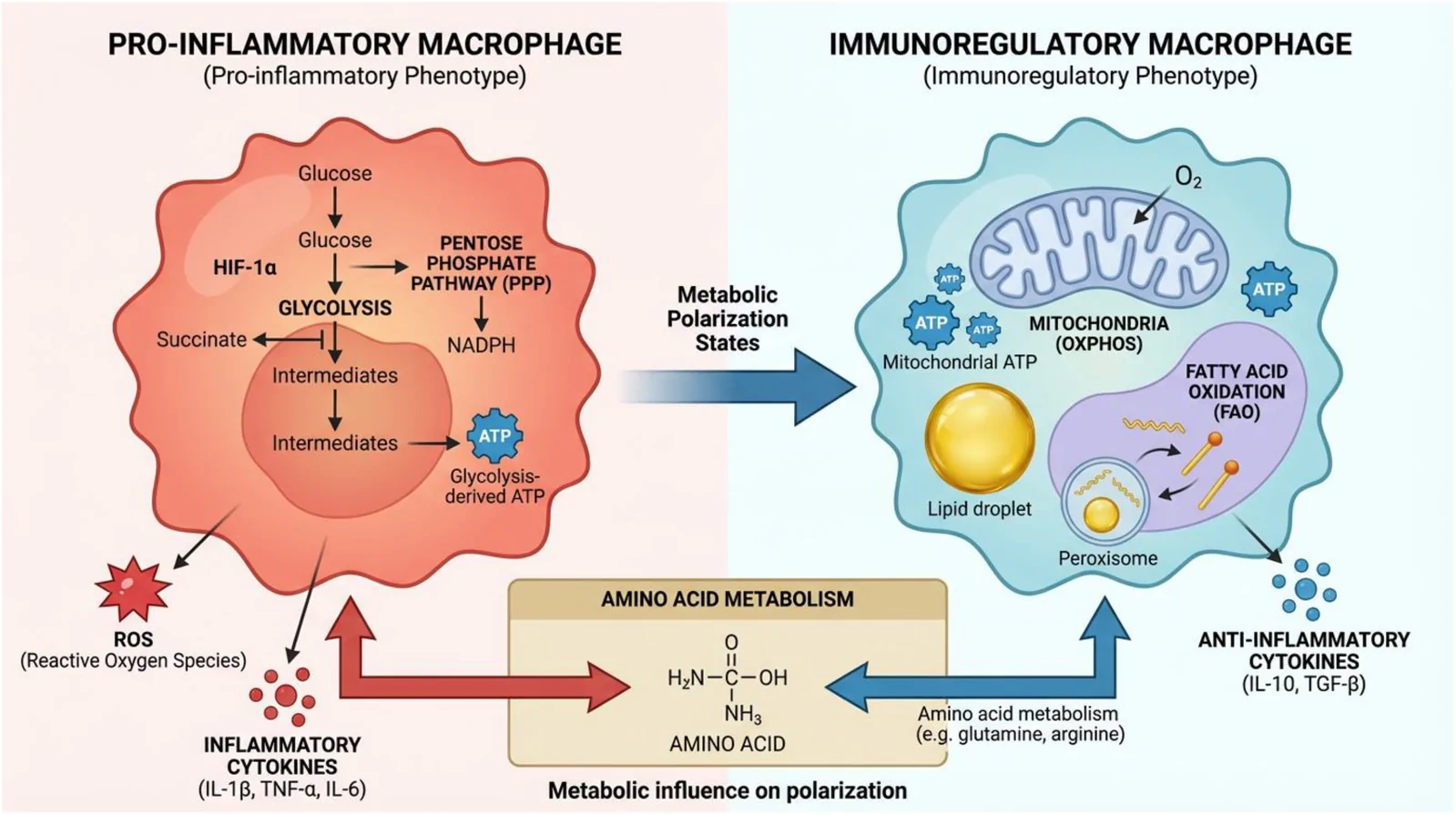
Core metabolic pathways controlling macrophage activation. Glycolysis, the pentose phosphate pathway, mitochondrial oxidative phosphorylation, and the TCA cycle coordinate macrophage metabolic programming and inflammatory responses.

## 3 Evolution of tumor-associated macrophage research

The historical development of tumor-associated macrophage (TAM) research spans more than a century, reflecting a steadily advancing understanding of the complex relationship between inflammation and cancer (19). A significant milestone has been achieved. Around the same time, a paradigm-shifting hypothesis emerged, suggesting that immune cells might not only suppress cancer but could also promote tumor growth (20). The advent of monoclonal antibody technology in the 1980s made possible the precise identification and isolation of macrophage subpopulations. In comparison, the development of genetically engineered mouse models in the 1990s revolutionized cancer immunology research. These advances have renewed interest in the tumor microenvironment (TME) in shaping macrophage behavior and influencing malignant progression (21).

Entering the 21st century, increasing evidence suggests that macrophage consumption can inhibit tumor growth and metastasis, although its impact on angiogenesis remains complex. Research conducted between 2012 and 2013 found that many macrophages in tissues originated from the embryonic yolk sac, thereby fundamentally altering the classic view of individual macrophage development (22, 23). In 2018, researchers provided a proof-of-concept demonstrating that TAMs could be reprogrammed to an anti-tumor phenotype, positioning macrophage regulation as a promising therapeutic strategy. In 2019, a CSF1R inhibitor for a specific type of cancer was approved, marking a significant milestone in the clinical advancement of targeted therapy. Recent studies have emphasized the close connection between TAM enrichment (23), immunosuppression, and poor clinical prognosis, underscoring its value as a biomarker and a therapeutic target in modern oncology (**Figure 3**).

**Figure 3:**
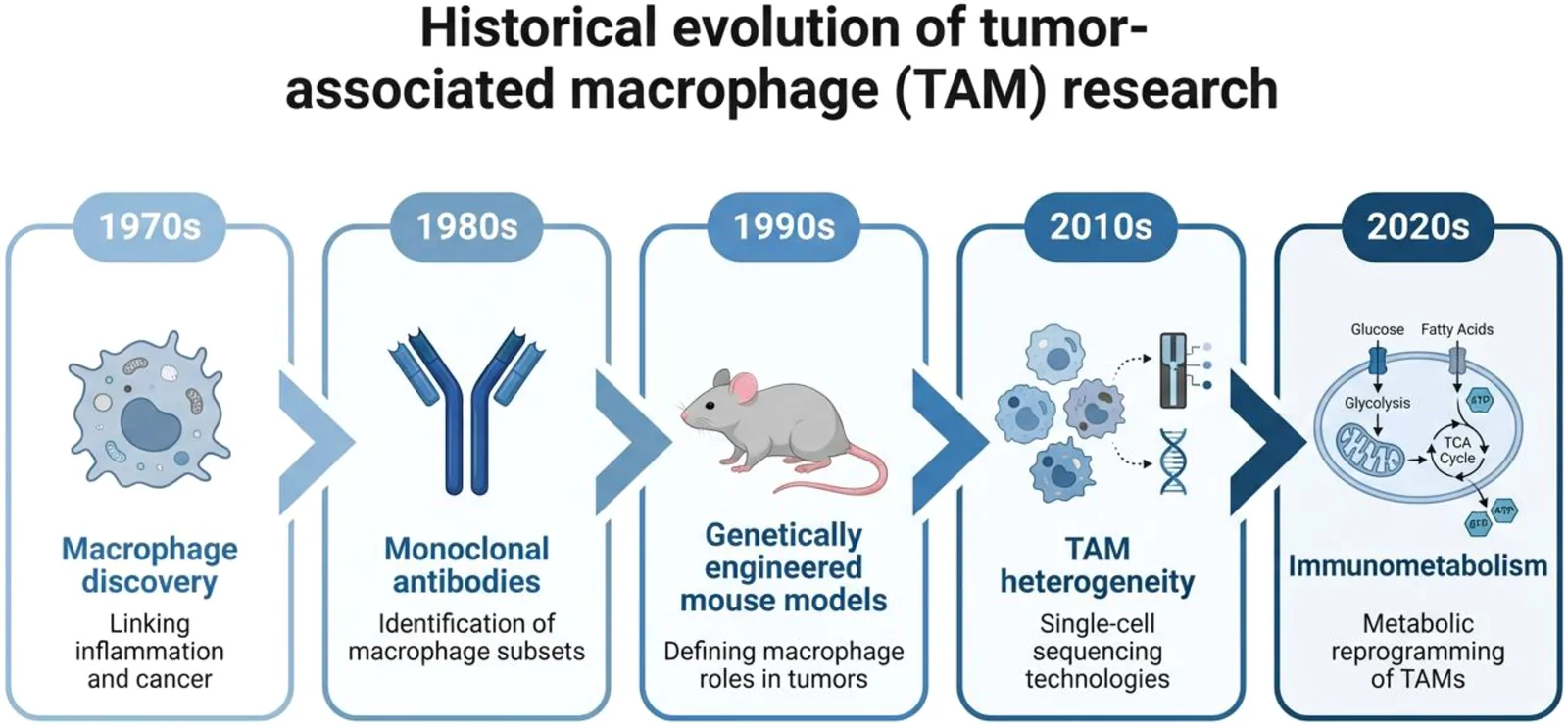
Historical development of tumor-associated macrophage (TAM) research. Major milestones highlight the evolving understanding of TAM biology, from early discoveries linking inflammation and cancer to modern insights into macrophage heterogeneity and therapeutic targeting.

### 3.1 Technological and conceptual advances in tumor-associated macrophage biology

Due to revolutionary technological advancements, the study of tumor-associated macrophages began in the 1970s (24). It was discovered that colony-stimulating factor (CSF)-1 is a key regulator of macrophage lineage commitment, and that specific molecular signals can control macrophage growth, marking a significant shift in understanding. With the development of monoclonal antibodies enabled by heterotumor technology in the 1980s, it became possible to identify and target different macrophage subsets *in vivo* precisely. By the 1990s, conditioned macrophage consumption systems, CSF-1-deficient (op/op) mice, and other transgenic mouse models had provided unprecedented insights into the development, survival, and tumor-promoting activities of macrophages (25, 26). High-dimensional flow cytometry, lineage tracing, and single-cell RNA sequencing (scRNA-seq) have revolutionized our understanding of TAM heterogeneity in the 21st century by identifying distinct macrophage subpopulations that differ based on tissue, tumor stage, and microenvironmental cues. Importantly, research since 2012 has shown that many tissue macrophages originate from embryonic yolk sac progenitor cells, challenging long-standing theories about their individual development (27). Reprogramming techniques such as CD40 agonists, CSF1R inhibitors, and metabolic regulators have ushered in a new era of macrophage-targeted immunotherapy, demonstrating that TAMs can be reprogrammed into anti-cancer phenotypes.

Importantly, many of these historical milestones preceded the recognition that macrophage function is metabolically constrained. Contemporary reinterpretation of TAM biology reveals that signaling pathways such as CSF-1/CSF-1R, STAT3, and hypoxia responses are tightly coupled to metabolic rewiring, including enhanced glycolysis, lipid uptake, and mitochondrial adaptation. These insights reposition TAM history within a modern immunometabolic framework.

## 4 Phenotypic and functional heterogeneity of tumor-associated macrophages

### 4.1 Classical and alternative macrophage activation states

Tumor-associated macrophages (TAMs) are a heterogeneous population characterized by varied phenotypic and functional states, generally categorized into M1-like and M2-like subsets. Macrophages start from bone marrow-derived monocytes that move into tissues, where they differentiate into resident or infiltrating populations. They engage in both innate and adaptive immunity, demonstrating extensive functional plasticity. M1 and M2 macrophages signify two primary activation states characterized by their triggering stimuli and immunological actions. Importantly, the classical M1/M2 framework represents a simplified conceptual model rather than a strict binary classification of macrophage states. Increasing evidence from single-cell RNA sequencing, spatial transcriptomics, and high-dimensional cytometry demonstrates that macrophages in tissues exist along a dynamic continuum of activation states shaped by metabolic conditions, cytokine exposure, and tissue-specific signals. In tumors, these approaches have revealed multiple tumor-associated macrophage subpopulations with distinct transcriptional, metabolic, and functional programs that the M1/M2 dichotomy cannot fully capture (28). Therefore, in this review, the M1/M2 terminology is used primarily as a heuristic framework to broadly describe pro-inflammatory versus immunoregulatory tendencies, while acknowledging that macrophage phenotypes *in vivo* are considerably more heterogeneous and context-dependent.

M1 macrophages are activated by interferon-γ (IFN-γ) and lipopolysaccharide (LPS), while M2 macrophages are stimulated by TH2-associated cytokines, including interleukin-4 (IL-4) and IL-13 (29, 30). M1-type macrophages release pro-inflammatory mediators and perform phagocytosis on pathogens and apoptotic cells, while M2-type macrophages produce anti-inflammatory cytokines. These cytokines can inhibit immune activation and promote tissue repair, remodeling, and the resolution of inflammation (31).

### 4.2 Antitumor and protumor functions of TAM subsets

These distinctions acquire particular significance within the TME. M1-like macrophages typically exert antitumor functions, whereas M2-like TAMs support tumor initiation, accelerate metastatic progression, inhibit the effector activity of T and B lymphocytes, promote angiogenesis, and assist in tissue reconstruction, collectively fostering a protumor microenvironment (32). M1-like macrophages restrict tumor growth and metastasis through three primary mechanisms: antibody-dependent cell-mediated cytotoxicity (ADCC), antibody-dependent cellular phagocytosis (ADCP), and the secretion of proinflammatory cytokines. They release IL-1β, IL-6, IL-12, and IL-23, as well as CXCL9 and CXCL10, and upregulate major histocompatibility complex (MHC) molecules, thereby strengthening antitumor immunity. Studies by Elsas et al. demonstrated that late-phase activation of M1-like macrophages is critical for controlling tumor outgrowth. M1 activation is also characterized by increased expression of CD68 and CD80, as well as elevated SOCS3, reinforcing their tumoricidal function (33).

By contrast, M2-like macrophages promote tumor proliferation and dissemination by secreting growth and immunosuppressive factors, including epidermal growth factor (EGF), platelet-derived growth factor (PDGF), transforming growth factor-β1 (TGF-β1), and basic fibroblast growth factor (bFGF). M2 macrophages further suppress CD8^+^ T cells, promote tumor proliferation and metastasis, and remodel the extracellular matrix (ECM) through matrix metalloproteinases (MMPs), serine proteases, and other enzymes. They also release proangiogenic factors necessary to maintain sufficient oxygen and nutrient supply for tumor expansion (34). The M2 category has expanded in recent years into four subtypes, M2a, M2b, M2c, and M2d, each defined by distinct cytokine environments and signaling pathways (35–37). M2a macrophages arise primarily in response to IL-4 and IL-13, whereas M2b macrophages are induced by immune complexes (IgG) in combination with Toll-like receptor (TLR) agonists (38). M2c macrophages are driven by glucocorticoids or IL-10–dependent macrophage colony-stimulating factor (M-CSF) signaling. M2d macrophages differ from the other subtypes in that they arise from previously polarized M1 macrophages and are induced by IL-6, TLR signaling, and A2 adenosine receptors (39).

### 4.3 Spatial organization of TAMs within the tumor microenvironment

TAMs are often the most abundant immune cell population in tumors, and their distribution correlates strongly with disease progression. In a study of 104 lung cancer patients, Zheng et al. reported that M1-type TAMs were more abundant than M2-type TAMs, with a significant enrichment of invasive-margin (IM) M2 TAMs (40). Both M1 and M2 TAMs were more prevalent in stromal rather than parenchymal regions. High-density M1 TAMs predicted improved overall survival, while M2 TAM density showed no significant association. Tumors located closer to M2 TAMs exhibited higher metastatic potential, and larger tumors were more frequently located near M2 IM-TAMs. Macrophage heterogeneity also varies by tissue of origin. In the breast, stromal macrophages (SMs) and ductal macrophages (DMs) form two significant tissue-resident populations. They identified multiple subgroups of tumor-associated macrophages (TAMs), which possess ecological characteristics distinct from those of macrophages present before tumor formation, and observed that TAM composition differed between proliferative and malignant lesions (41). Compared with proliferative lesions, CD11b expression was significantly increased in malignant lesions, consistent with the trend observed in the pre-tumor stage.

### 4.4 Functional plasticity and dynamic reprogramming of TAMs

TAMs exhibit extensive heterogeneity in both phenotype and function (42, 43). Their roles vary depending on local microenvironmental signals, enabling them to either support antitumor immunity or promote tumor progression (44, 45). While M1 and M2 macrophages represent two ends of an activation continuum, macrophages can transition between these states in response to environmental cues, as shown in **Figure 4**. As a result, multiple studies have explored strategies to pharmacologically reprogram protumor M2 macrophages into an antitumor M1 phenotype, aiming to improve cancer immunotherapy outcomes (46).

**Figure 4:**
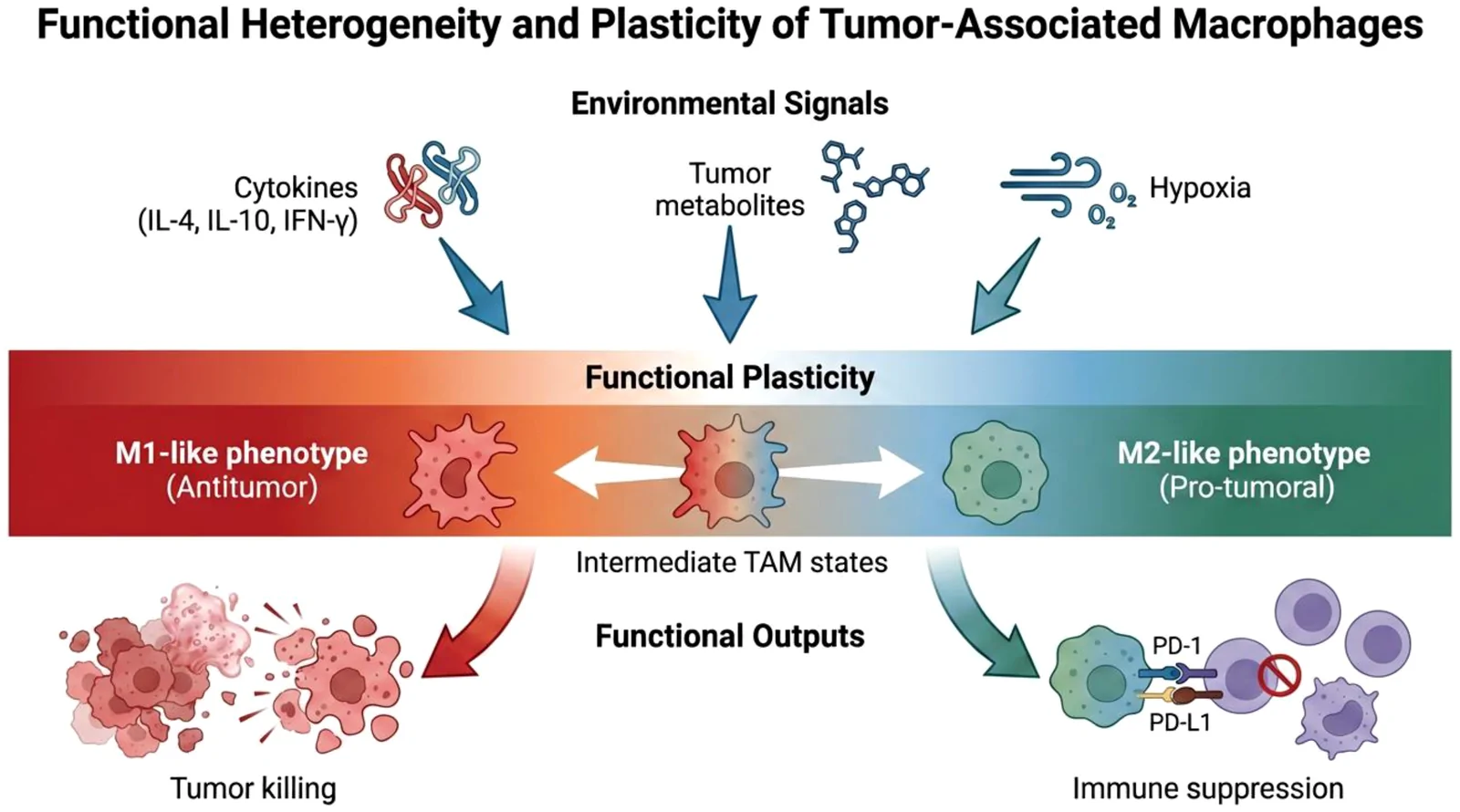
Heterogeneity and plasticity of tumor-associated macrophages. TAMs exist along a dynamic spectrum of activation states shaped by signals from the tumor microenvironment.

## 5 Immunometabolic mechanisms of TAM-mediated immune suppression

Within the tumor microenvironment (TME), multiple tumor-derived factors act in concert to suppress antitumor immunity by shaping macrophage phenotype and function. These regulatory effects occur through tightly interconnected signaling and metabolic networks in which cytokine signaling, nutrient availability, and metabolic competition collectively determine macrophage immunosuppressive activity. Cytokines, such as IL-10, and lipid mediators, such as prostaglandin E2 (PGE2), released by tumor cells, can skew monocytic MDSCs toward an M2-like TAM phenotype. This process can be reversed by inhibiting prostaglandin synthesis through COX-2 blockade (47–49). Furthermore, IL-1 has been shown to elevate TET2 expression, a DNA demethylase, thereby sustaining immunosuppressive TAM activity in both human and murine melanoma systems (50). M2-type tumor-associated macrophages significantly suppress the adaptive immune response by upregulating multiple immune checkpoint ligands. They express high levels of programmed death ligand 1 (PD-L1), which binds to PD-1 on T cells, leading to cell exhaustion and a weakened anti-tumor effector capacity. They also synthesize Fas ligand (FasL), which binds to Fas on T cells, triggering cell apoptosis and promoting local immune tolerance (51). Additionally, increased expression of HLA-G (a non-classical MHC-I molecule) further inhibits the cytotoxicity of natural killer (NK) cells and limits T cell responses, thereby strengthening the immunosuppressive environment (52). Tumor-associated macrophages also impose metabolic restrictions on T cells. Arginase activity reduces extracellular arginine, an essential resource for T cell proliferation. At the same time, indoleamine 2,3-dioxygenase (IDO1) converts tryptophan into kynurenine derivatives that promote the formation of regulatory T cells (Tregs) and immunosuppression (53). The M2 type tumor-associated macrophages also express many checkpoint molecules, including B7-H4, PD-L1, and VISTA, all of which promote T cell suppression. Therapeutic antibodies targeting these pathways have been shown to effectively alleviate the immunosuppressive effects of TAM (50). Although immunosuppressive TAMs are often described as “M2-like,” recent single-cell studies indicate that these populations comprise multiple transcriptionally and metabolically distinct macrophage states that occupy intermediate positions along the macrophage polarization continuum.

Cytokine secretion further amplifies TAMs’ immunosuppressive effects. IL-10 produced by TAMs impairs dendritic cell maturation. It hinders the priming of effective antitumor T-cell responses, whereas TGF-β1 supports the persistence and activity of regulatory T cells, thereby deepening immune suppression (**Figure 5**) (54). In models of multiple myeloma (MM), TAM-derived IL-1β and IL-18 promote the accumulation of myeloid-derived suppressor cells (MDSCs), thereby impairing CD8^+^ T-cell function (55). Notably, deletion of IL-18 in mice prevented MM progression by restoring CD8^+^ T-cell activity, and elevated bone marrow IL-18 levels in MM patients correlated with poorer survival (56).

**Figure 5:**
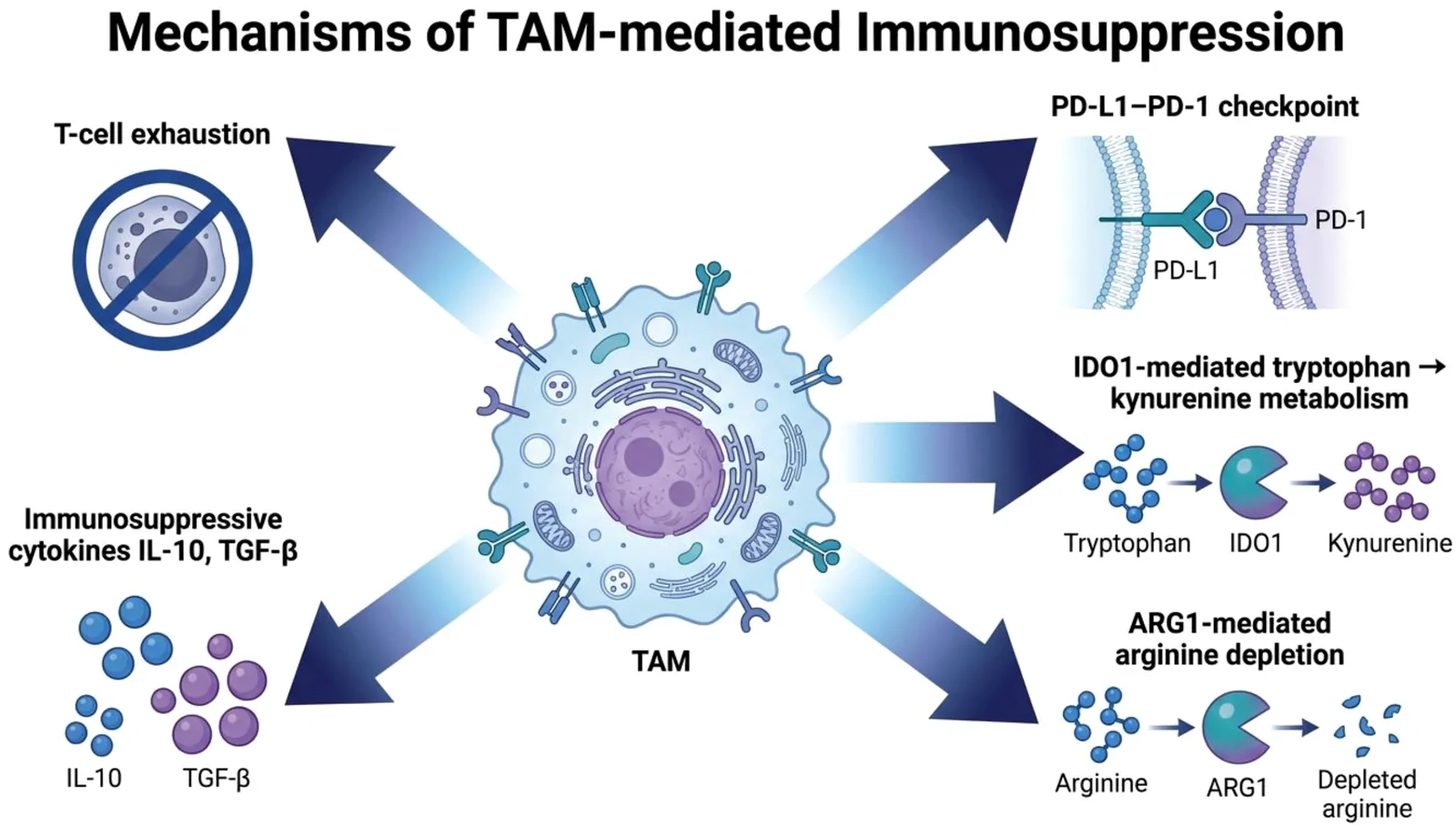
Immunometabolic mechanisms of TAM-mediated immune suppression. Tumor-derived cytokines and metabolites promote immunosuppressive TAM phenotypes that inhibit antitumor T-cell responses.

### 5.1 Metabolic and cytokine-dependent pathways driving TAM-induced immune suppression

Through metabolic and cytokine-driven processes, TAMs not only participate in checkpoints but also exert significant immunosuppressive effects (57). Extracellular arginine is an essential amino acid for T cell proliferation and the formation of the T cell receptor (TCR) chain, and it is consumed by arginase-1 (a key pathway). Meanwhile, tryptophan is metabolized by IDO1 into kynurenine, a metabolite that inhibits the effector function of CD8 T cells and promotes Treg cell proliferation. Due to these metabolic limitations, T cells are unable to maintain their anti-tumor activity in this “nutrition-restricted” environment. TAM-derived cytokines, including IL-10, TGF-β, IL-18, and IL-1β, further enhance immunosuppression, reduce antigen presentation, restrict dendritic cell maturation, and promote the recruitment of regulatory T cells. The IL-18 and IL-1β released by TAM have been shown to increase myeloid-derived suppressor cells (MDSCs), thereby amplifying the immunosuppressive network in multiple myeloma and melanoma models (58). In hypoxic tumor regions, TAM employs a transcriptional program that emphasizes glycolytic ATP synthesis and the expression of immunosuppressive molecules. These metabolic and cytokine-mediated pathways are the most prominent. T cell function can be restored and immunotherapy responsiveness improved by reprogramming macrophage metabolism, blocking the IL-10/TGF-β axis, or reducing IDO1.

## 6 Signaling networks regulating TAM function in the tumor microenvironment

Tumor-associated macrophages (TAMs) have complex and often opposing functions in the tumor microenvironment (TME). M1-type TAMs can enhance the inflammatory response and directly eliminate malignant cells, but M2-type TAMs usually modify the microenvironment to promote tumor growth, angiogenesis, immunosuppression, and metastasis (**Figure 6**). This functional difference mainly stems from their polarization state, which determines whether TAMs exert anti-tumor effects or promote tumor growth.

**Figure 6:**
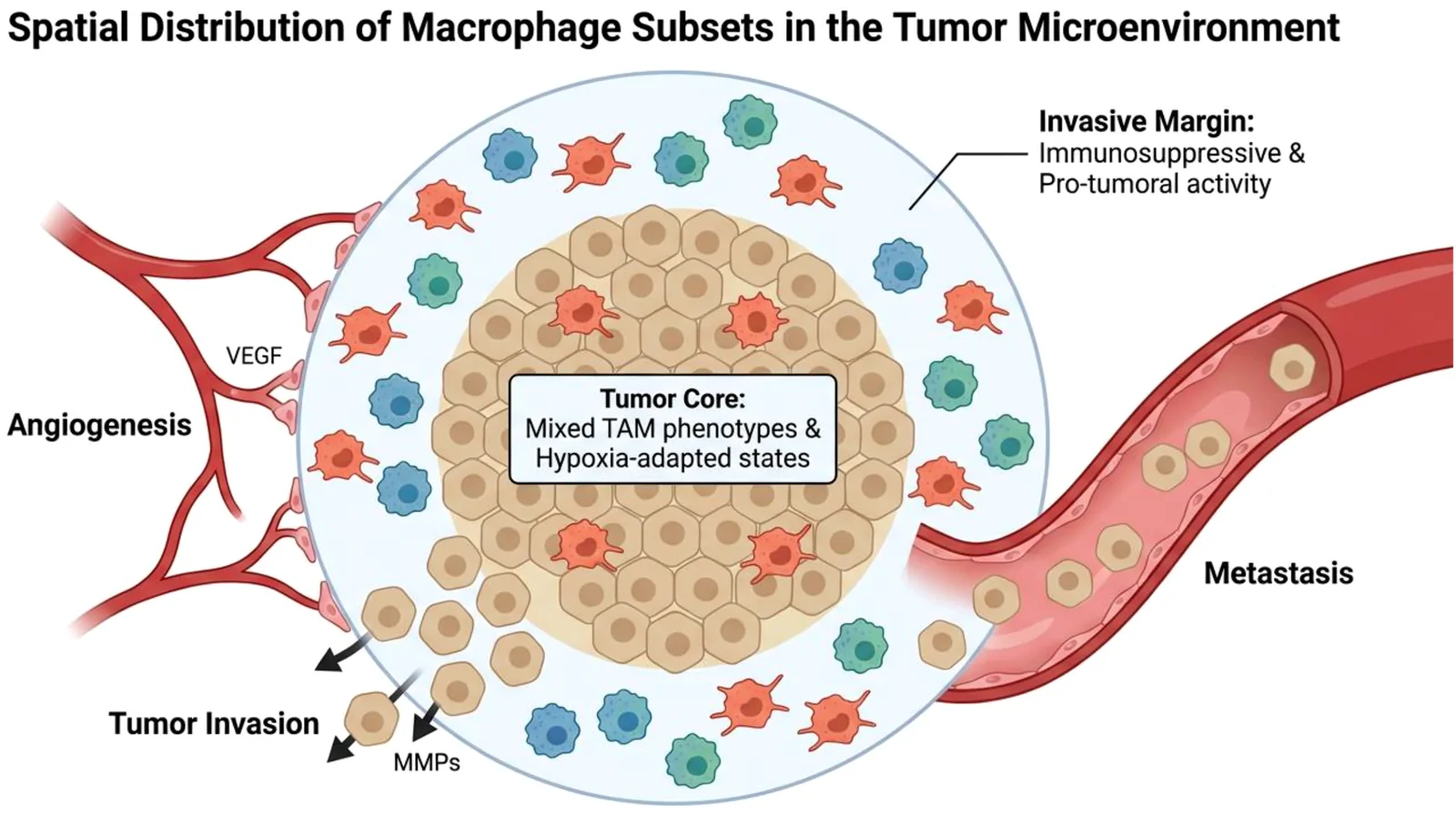
Spatial distribution of macrophage subsets in tumors. Distinct macrophage populations localize to distinct tumor regions, contributing either to antitumor immunity or to tumor progression.

### 6.1 Core signaling pathways governing TAM activation and polarization

Tumor-associated macrophages (TAMs) influence tumor biology through signaling pathways that regulate inflammation, angiogenesis, immune evasion, and extracellular matrix (ECM) remodeling. These interrelated pathways determine macrophage behavior in the tumor microenvironment and their role in tumor suppression or progression. Macrophage activation is highly flexible and depends on cytokine exposure, intercellular interactions, and signals from the local microenvironment. Pathways related to nuclear factor-κB (NF-κB) and the rapamycin target site (mTOR) play a key role in determining macrophage phenotype. Under sufficient stimulation, tumor-associated macrophages (TAMs) produce large amounts of reactive oxygen species (ROS), activate NF-κB, and initiate downstream transcriptional programs, thereby influencing tumor cells and leukocytes, continuously triggering chronic inflammation within the tumor. Clec4e is an upstream regulator that can activate Syk, thereby initiating NF-κB signaling and enhancing tumor-associated macrophage (TAM) pro-tumor activity (59). Therapeutic inhibition of TNF-α has shown potential for counteracting this pathway (60). Conversely, IFN-γ can diminish TAM accumulation and reverse their immunosuppressive and tumor-promoting features (61). NF-κB also contributes to tumoricidal responses by modulating macrophage cytotoxicity (62).

The STAT pathway is also another key factor in the regulation of inflammation. Cytokines produced by the tumor-associated microenvironment (TAM), such as IL-6 and IL-10, activate signal transducer and activator of transcription 3 (STAT3) by phosphorylating Janus kinase (JAK). The dimerization of STAT3 ultimately drives intracellular metastasis and induces the expression of cyclin D1, VEGF, and MMP genes, which are critical for angiogenesis, invasion, and tumor cell proliferation. STAT3 also weakens T cell immune function by increasing programmed death ligand 1 (PD-L1) expression. In studies on ovarian cancer, it has been shown that TAMs expressing PD-L1, a STAT3-dependent molecule, can bind to PD-1, leading to T cell inactivation (63). Additionally, survival genes, including Bcl-xL and Mcl-1, are expressed more strongly when STAT5 is activated. To enable tumor growth and metastasis, tumor vascularization is crucial for delivering oxygen and nutrients. TAMs regulate this process through the pathways of vascular endothelial growth factor (VEGF) and hypoxia-inducible factor 1α (HIF-1α). Hypoxia increases HIF-1α expression in tumor-associated macrophages, which, in turn, encourages the generation of vascular endothelial growth factor (VEGF), as the macrophages directly stimulate endothelial cells by releasing VEGF. Due to these processes, anti-angiogenic drugs such as Avastin and Sorafenib can prevent tumor development.

### 6.2 TAM-driven extracellular matrix remodeling and tumor invasion

For cancer cells to invade and metastasize, they must modify or degrade the extracellular matrix. TAMs significantly contribute to this process by activating matrix metalloproteinases (MMPs). Cytokines secreted by TAMs, including TNF-α, IL-1β, and TGF-β, upregulate MMP production. TAM-derived IL-1β, for instance, enhances the expression of MMP-2 and MMP-9 (64). Proteases such as Cathepsin B can further activate latent MMP precursors. Once active, MMPs dismantle ECM structures, enabling tumor cells to migrate and promoting the formation of new blood vessels, known as neovascularization. This TAM-driven ECM remodeling is well-documented in breast cancer.

### 6.3 Immune checkpoint and immunosuppressive signaling pathways in TAMs

Tumor-associated macrophages (TAMs) can inhibit T cell activity by expressing PD-L1, which binds to PD-1 on T cells, thereby inhibiting T cell activation and cytotoxicity. In ovarian cancer, PD-L1 derived from TAMs has been shown to directly induce T cell exhaustion. Tumor-associated macrophages (TAMs) may express PD-1, and their activation inhibits the phagocytic function of macrophages. Inhibiting PD-1 can enhance TAM-mediated phagocytosis and prolong survival in mice with colon cancer models (65). These interactions highlight the role of TAMs in promoting immune evasion. TAMs also regulate the immune response through cytotoxic T lymphocyte-associated protein 4 (CTLA-4). TAM expression of CTLA-4, combined with B7.1 and B7.2 on T cells, inhibits T cell growth and activation (66). Cytokines secreted by tumor-associated macrophages, including IL-10 and TGF-β, act synergically with CTLA-4 to enhance immunosuppression and promote tumor progression. In addition to these main pathways, TAMs employ various secondary strategies to improve the survival and spread of tumor cells. TAMs promote macrophage survival via the PI3K/Akt signaling pathway and inhibit apoptosis (67). The Ras/ERK signaling pathway helps their proliferation and differentiation. The interaction between macrophages and the extracellular matrix (mediated by integrins) controls adhesion, localization, and tissue remodeling. Moreover, the CSF-1/CSF-1R signaling is crucial for the growth and maintenance of tumor-associated macrophages (TAMs), making it a key axis in TAM biology (68).

These pathways form a complex regulatory network that enables tumor-associated macrophages to manage tumor development, angiogenesis, immunosuppression, and metastasis. The complexity of their signals also enhances resistance to certain cancer treatment modalities. A comprehensive understanding of these pathways at the intersection of development can reprogram TAM activity and restore effective anti-tumor immune therapy, which is crucial, as shown in **Figure 7**.

**Figure 7:**
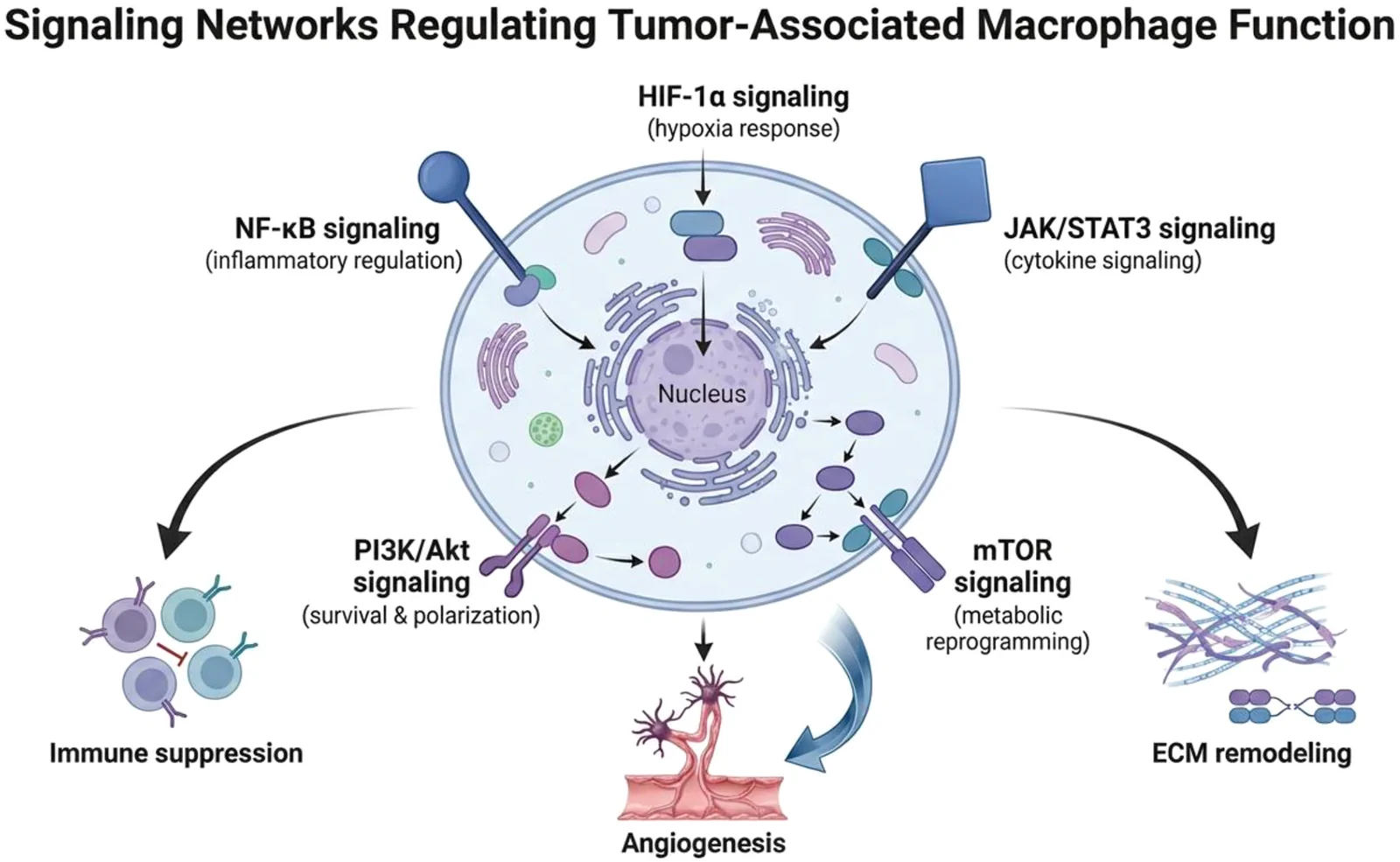
Signaling pathways regulating TAM activation and function. Key signaling networks—including NF-κB, JAK/STAT, PI3K/Akt, and HIF-1α—control TAM polarization and tumor-promoting activities.

## 7 Metabolic crosstalk between tumor cells and tumor-associated macrophages

Compared to normal cells, tumor cells exhibit distinct glucose, lipid, and amino acid metabolism, which promotes tumor growth and enhances their ability to adapt to hypoxic and nutrient-deficient environments. A prominent example is the Warburg effect, in which tumor cells prioritize aerobic glycolysis even when sufficient oxygen is available. The increase in lactic acid produced by this metabolic change is output by transporters such as MCT4, which acidifies the TME and reduces the function of immune cells (69). A powerful signaling chemical substance that promotes immunosuppressive polarization of T cells is lactic acid (70, 71). Tumors also significantly alter lipid metabolism. Membrane biosynthesis, redox maintenance, and oncogenic signaling all depend on increased fatty acid synthesis, arachidonic acid conversion, and cholesterol and sphingolipid metabolism (72–74). Lipid metabolites link immune and metabolic control in the TME by directly promoting TAM polarization and immunosuppression (75, 76). Tumor cells exhibit excessive activation of amino acid metabolism, particularly in pathways involving arginine, glutamine, and tryptophan. These amino acids alter the TAM phenotype, acting as both biosynthetic precursors and regulatory metabolites to modulate anti-tumor immunity. Combined, these changes create a metabolic environment that promotes the transformation of Tams into a state that supports tumor growth. These observations highlight that metabolic crosstalk between tumor cells and macrophages is a central determinant of TAM functional programming within the tumor microenvironment.

However, many mechanistic insights into TAM metabolic reprogramming, including lactate-driven polarization, lipid metabolic remodeling, and amino acid–dependent immune regulation, have been established primarily in murine tumor models. Differences in metabolic regulation between mouse and human macrophages, particularly in nitric oxide production and lipid metabolism, may influence the extent to which these pathways operate in human tumors (77). Recognizing these interspecies differences is essential for interpreting preclinical findings and designing translational immunometabolic therapies.

### 7.1 Metabolic interactions between tumor cells and TAMs

In addition to promoting cancer cell growth, tumor cell metabolic reorganization also exerts a powerful immunomodulatory effect. Tumor cells alter Tams’ function by secreting metabolic intermediates, thereby promoting angiogenesis, metastasis, and immune escape. An important metabolite that affects TAM behavior is lactic acid (78, 79). Tumor cells exhibit high glycolytic flux, reduced mitochondrial activity, and produce a large amount of acid, placing macrophages in a state of nutritional competition and rendering them more akin to M2 cells (80). By reducing cytotoxic immune responses and stabilizing HIF-1α in tam, lactic acid accumulation strengthens immunosuppression, while promoting VEGF synthesis and angiogenesis (81). Lactic acid stimulates PKA/CREB signaling, activates transcription factors, including Zeb1, and promotes tumor development and tam-mediated chemotherapy resistance in acidic TMEs (67). By differentially activating pathways such as the KRAS/MAPK pathway, the spatial lactate gradient further regulates different TAM subpopulations (82). In cervical cancer and glioma, lactic acid-driven transcriptional and epigenetic alterations can improve M2 polarization and accelerate the development of malignant tumors (83). Tumor cell-derived lipids also have a significant impact on tam. When macrophages absorb unsaturated fatty acids, the M2-like phenotype is enhanced, and unsaturated fatty acids bind to FABP5 and activate PPARγ (84). PGE2 and other arachidonic acid metabolites reduce M1 bioenergy and induce M2 polarization through the cAMP-CREB and EP2/EP4 pathways, thereby promoting immunosuppressive TAM function (85). Furthermore, PGE2 enhances immune evasion by upregulating PD-L1 expression on TAMs. Tumor-derived 27-hydroxycholesterol increases M2 polarization and activates PPARγ in TAMs of hepatocellular carcinoma. Through endoplasmic reticulum stress and STAT3-XBP1 activation, tumor-derived sphingolipids such as S1P and glycosylceramides can also promote the TAM phenotype that supports tumors (86).

Amino acids further influence TAM behavior. Although the glutamine metabolite α-α-ketoglutarate supports M2-related epigenetic programs, tumor-driven glutamine depletion hinders M2 polarization (87). Glutamate metabolism is improved in Tams, and its M2 polarization is driven by the upregulation of GLS1 and the release of GLS1-rich vesicles by HER2-positive gastric cancer cells. Aspartic acid produced by glutamine promotes the M2 phenotype by facilitating EIF5A-dependent HIF-1α translation (88). In malignant tumors, arginine metabolism tends to produce polyamines, which inhibit CD8+ T cell activation via epigenetic mechanisms, thereby driving M2 polarization (89). Through these metabolic interactions, TAMs are positioned as key mediators of tumor immune evasion and development, which are directly driven by tumor cell metabolism **Figure 8**.

**Figure 8:**
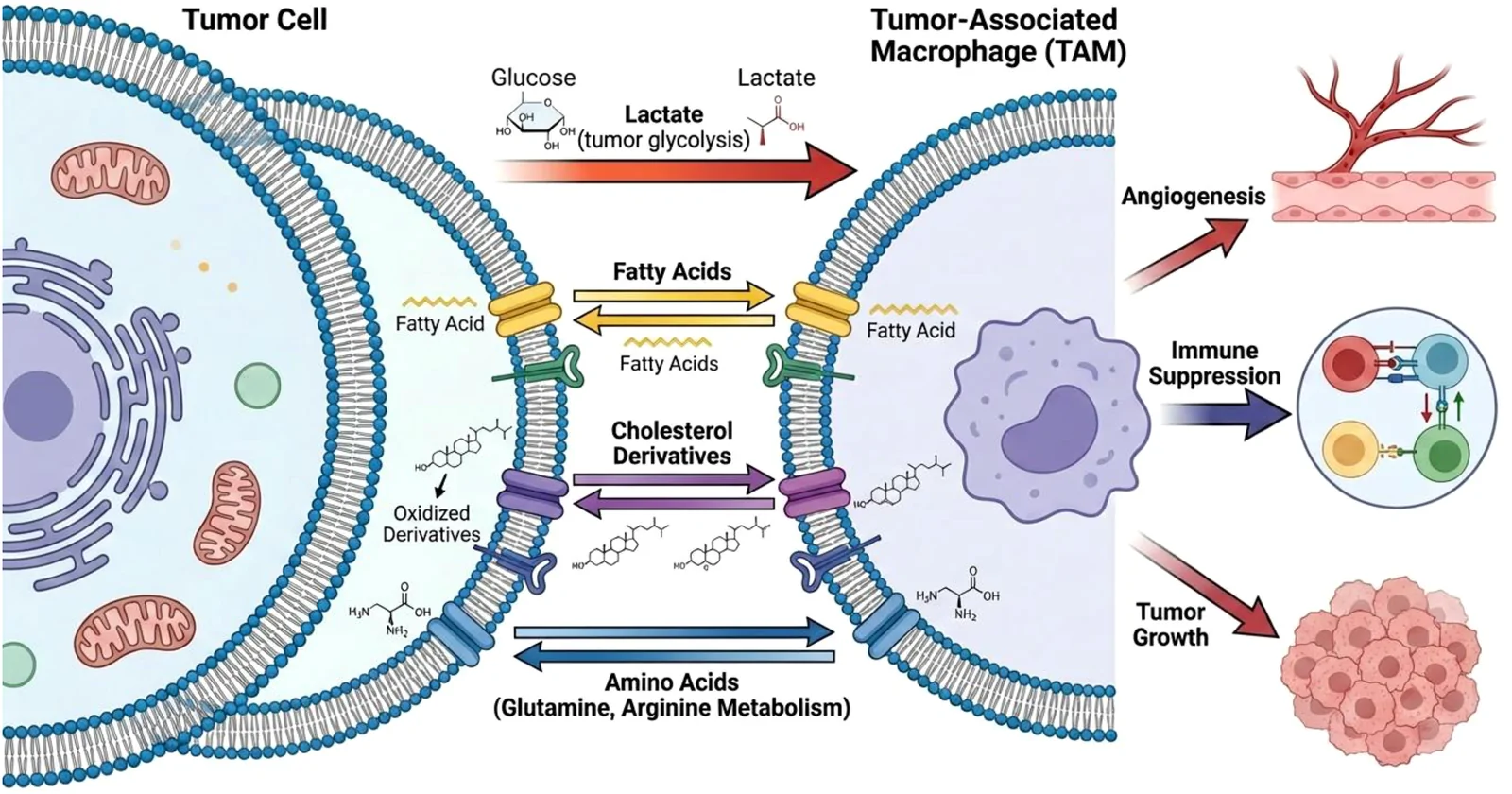
Metabolic crosstalk between tumor cells and TAMs. Tumor-derived metabolites such as lactate, lipids, and amino acid products reshape TAM metabolism and promote immunosuppressive functions.

### 7.2 Immunometabolic remodeling of TAMs during tumor progression

Tumor behavior, as influenced by TAM, TME, and experience, is shaped by numerous metabolic remodeling events. In a polarized state, their distinct sugar metabolisms are evident: M1 macrophages support ROS production via glycolysis, killing microorganisms, and exhibit vigorous PPP activity, whereas M2 macrophages depend on OXPHOS and mitochondrial function (90). Macrophage metabolic remodeling in tumors is not merely a consequence of environmental stress but rather an active regulatory layer that controls macrophage phenotype. Key metabolic checkpoints integrate nutrient availability with transcriptional and epigenetic programs that stabilize TAM polarization states. For example, glycolytic regulators such as PDK1 and HIF-1α promote inflammatory or hypoxia-adapted macrophage programs by controlling pyruvate flux and mitochondrial respiration (91). In contrast, lipid-sensing pathways involving PPARγ and fatty acid–binding proteins (FABPs) regulate transcriptional networks associated with immunosuppressive TAM phenotypes. Similarly, amino acid–derived metabolites, including α-ketoglutarate and polyamines, can influence macrophage epigenetic landscapes via chromatin-modifying enzymes, thereby stabilizing protumoral macrophage states. These mechanisms illustrate those metabolic pathways function as signaling hubs rather than simple energy sources, linking nutrient competition within the tumor microenvironment to macrophage functional plasticity. Necessary enzymes, such as SHK, are regulated to control PPP activity and distinguish between the immunosuppressive and inflammatory functions of macrophages. Similarly, PDK1 alters macrophage glucose metabolism by regulating OXPHOS balance and glycolysis (92). Tams related to glioblastoma release HEXB, which increases the expression of glycolytic genes and tumor invasiveness by activating YAP1 in tumor cells and stabilizing HIF-1α. Hedgehog signaling inhibition can reverse Oxphos-dependent M2 polarization and restore immune function, whereas hypoxic TMEs reduce TAM glucose uptake, increase glucose availability to tumor cells, and promote cancer progression (93). TAM lipid metabolism also affects tumor development. Lipid droplet accumulation caused by Caspase-1-mediated PPARγ processing promotes tumor development (94). Members of the FABP family have contradictory effects: A-FABP promotes metastasis by activating NF-κB/miR-29b/IL-6/STAT3 signaling, while E-FABP enhances anti-cancer immunity by increasing IFN-β production (95). In breast cancer, Tams expressing COX-2 synthesize eicosanoids to stimulate invasion, endocrine resistance, EMT, and survival (96). Another factor is cholesterol homeostasis: The 27HC produced by tam can promote cancer formation, while ABCG1 deficiency leads to M1-like activation and inhibits tumor growth (97). Depending on the cell type, MGLL can exert both tumor-promoting and tumor-suppressing effects, underscoring the complexity of lipid metabolism in the TME (98).

The function of TAM is further regulated by amino acid metabolism. Tumors reduce NO production and weaken anti-tumor immunity by redirecting arginine fluxes to polyamine synthesis (99). Her2-positive breast cancer can be inhibited by sepapterin treatment, which can restore the production of NO when glutamine synthase is inhibited. Additionally, tam, succinic acid, glycolysis, and T cell recruitment all increase (100). The activation of AHR in TAM by tryptophan decomposition metabolites can promote immunosuppression. Reducing dietary tryptophan or inhibiting microbial metabolites can improve immune responses (101).

### 7.3 Metabolic regulation of macrophage cell death: focus on ferroptosis

Beyond regulating activation and polarization, macrophage metabolism critically influences programmed cell death pathways, with ferroptosis emerging as a key immunometabolic mechanism (102). Ferroptosis is an iron-dependent form of regulated cell death driven by lipid peroxidation and oxidative stress, tightly linked to lipid metabolism, mitochondrial function, and redox homeostasis (103). Macrophages exhibit unique sensitivity to ferroptotic signals due to their roles in iron handling, lipid processing, and ROS production (104). Alterations in fatty acid composition, particularly the accumulation of polyunsaturated fatty acids within membrane phospholipids, increase susceptibility to lipid peroxidation. Dysregulation of glutathione metabolism and impaired glutathione peroxidase 4 (GPX4) activity further predispose macrophages to ferroptotic stress (105). Beyond antioxidant defense, ferroptosis sensitivity in macrophages is closely linked to broader immunometabolic programs operating within the tumor microenvironment. Lipid metabolism represents a central determinant of ferroptotic vulnerability because the accumulation of polyunsaturated fatty acids within membrane phospholipids provides substrates for lipid peroxidation. Tumor-associated macrophages frequently undergo lipid metabolic reprogramming characterized by increased fatty acid uptake, lipid droplet formation, and activation of PPARγ-dependent transcriptional programs (106). These changes can alter phospholipid composition, thereby influencing susceptibility to ferroptotic damage. At the same time, macrophage redox balance, regulated by pathways controlling mitochondrial respiration, reactive oxygen species production, and NADPH availability, further modulates lipid peroxidation dynamics. Iron metabolism, including macrophage-mediated iron recycling and storage through ferritin and transferrin systems, also contributes to the regulation of ferroptosis by controlling the intracellular labile iron pool that drives oxidative reactions.

These mechanistic connections suggest that ferroptosis represents an important link between macrophage metabolic programming and immune regulation in tumors. Therapeutically, strategies that modulate lipid metabolism, disrupt antioxidant defenses, or alter iron availability may selectively influence TAM survival and function. For example, inhibition of GPX4 activity or induction of lipid peroxidation has been proposed as a strategy to promote ferroptosis in immunosuppressive macrophage populations, potentially enhancing antitumor immunity (107). Conversely, regulating macrophage ferroptosis may also prevent excessive inflammatory damage in non-cancer contexts. Integrating ferroptosis-targeting approaches with metabolic inhibitors or immune checkpoint blockade may therefore represent a promising direction for future immunometabolic therapies. Importantly, ferroptosis intersects with macrophage immune function. Ferroptotic macrophages release damage-associated molecular patterns (DAMPs) and lipid mediators that reshape local immune responses, influencing inflammation, antimicrobial defense, and tumor immunity (108). Conversely, macrophages can regulate ferroptosis in neighboring cells by modulating iron availability, lipid transport, and oxidative balance within tissue microenvironments.

Recent evidence suggests that targeting ferroptosis-associated metabolic pathways may reprogram macrophage function, offering novel therapeutic opportunities (109). Manipulating lipid metabolism, iron handling, or antioxidant capacity in macrophages holds promise for modulating immune responses in cancer, infection, and inflammatory diseases.

### 7.4 Therapeutic targeting of immunometabolic pathways in tumor cells and TAMs

Therapeutic targeting of immunometabolic pathways is increasingly recognized as a strategy to reshape macrophage function rather than inhibit tumor cell metabolism. Many metabolic inhibitors influence both tumor cells and immune cells, thereby altering the competitive nutrient landscape within the tumor microenvironment (110). Three major mechanistic intervention strategies have emerged: (1) modulation of tumor-derived metabolites that drive TAM polarization (e.g., lactate or lipid mediators), (2) direct reprogramming of macrophage metabolic pathways such as glycolysis, fatty acid oxidation, and amino acid metabolism, and (3) combination approaches integrating metabolic inhibitors with immunotherapies such as immune checkpoint blockade. Focusing on these mechanistic categories helps clarify how metabolic interventions can shift TAMs from immunosuppressive to immunostimulatory states, thereby enhancing antitumor immunity (111).

The development of drugs targeting metabolic pathways offers broad prospects for enhancing anti-tumor immunity. LDHA inhibitors, including oxamic acid and machilin A, can restrict tumor development, reduce lactic acid production, and promote M1 polarization in TAMs (112). Shikonin promotes necrosis and ptosis and reverses treatment resistance (113), while PFK158 inhibits glycolysis and triggers apoptosis. Benserazide and C-02 are examples of HK2 inhibitors and degraders, which can effectively reduce glucose metabolism and overcome drug resistance (114). Although tumors with high MCT4 are drug-resistant, AZD3965 inhibits MCT1, reduces lactate output, and synergizes with radiotherapy (115, 116). IDH inhibitors help mutant cancers restore normal metabolic and epigenetic states (117). Tumor bioenergy is inhibited by CPI-613 and lonidamine, which interfere with mitochondrial metabolism (118). When it comes to lipid metabolism, there are more treatment options. FASN inhibitors, such as TVB-2640, have demonstrated clinical potential and significant anti-cancer effects (119). Farnesyltransferase inhibits lipid production mediated by SREBP, while terbinafine inhibits SQLE and suppresses liver and breast malignancies (120). BPTES and CB-839 are examples of glutamine metabolism inhibitors that restrict tumor growth and disrupt redox homeostasis (121). On the other hand, nanosystems combined with multiple metabolic inhibitors can cause strong immune activation. DFMO inhibits tumor growth by disrupting the tumor-associated macrophage (TAM)- associated polyamine axis.

Tailored intervention measures for TAMs also show the same prospects. arenbufagin repolarizes tam to the M1 phenotype by inhibiting LOX-1 and activating ABCA1 (122). Celecoxib and other COX-2 inhibitors increase IFN-γ production, reduce PD-L1 expression, and synergize with PD-1 inhibition (123). Metformin inhibits the COX-2/PGE2 axis and limits TAM invasion. EI-05 promotes TAM-mediated tumor suppression and increases E-FABP activity (124). Etomoxicillin inhibits fatty acid oxidation and reduces tumor cell migration, while decitabine reverses M2 polarization by blocking FAO. Glufosinate-ammonium can enhance anti-tumor immunity and reduce glutamine levels (125). When PI3Kγ is inhibited, ARG1 decreases, iNOS activity increases, and macrophage tumor-killing function improves (126). Due to its systemic effects, PEG-BCT-100 must be used with caution even though it consumes extracellular arginine. Ido1-mediated tryptophan metabolism is the focus of Epagata. However, to achieve a lasting effect, dual inhibition of IDO1/TDO may be necessary (127). Chloroquine promotes M1 polarization by altering macrophage metabolism, while 2-DG inhibits glycolysis and prevents EMT (128). Strategies including metabolic targets are becoming increasingly popular. The glutamine antagonist prodrug JHU083 reduces the possibility of metastasis by reprogramming tumor cells and TAMs (129). Simvastatin repolarizes TAMs through the LXR/ABCA1 pathway, disrupts lipid rafts, and resensitizes drug-resistant cancer cells to paclitaxel (130, 131). The nanoparticle version further enhances delivery and therapeutic effects (**Figure 9**).

**Figure 9:**
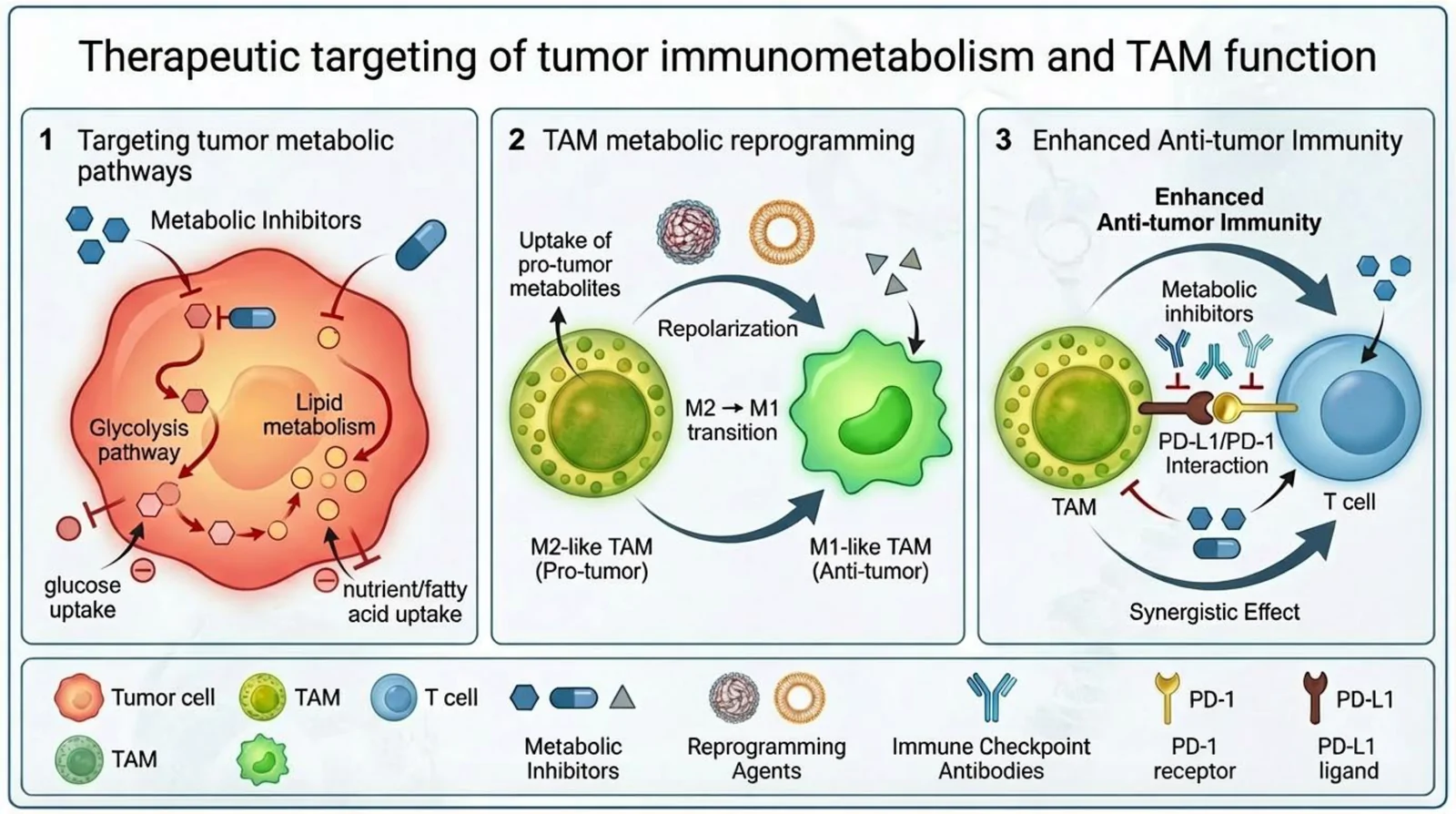
Metabolic therapeutic strategies targeting tumor cells and TAMs. Pharmacological modulation of glycolysis, lipid metabolism, amino acid pathways, and mitochondrial metabolism can reprogram TAMs and enhance antitumor immunity.

It is now known that macrophages are essential targets for immunotherapy. The CAR-macrophage technology based on adenovirus vectors can not only provide functional CAR expression but also induce M1 polarization, thereby promoting persistent tumor suppression and reducing metastasis (132). The fact that targeting Dectin-1 + tam can activate T cells and synergistically enhance tumor death when combined with PD-L1 inhibition highlights the potential of combined therapy (133).

Despite encouraging preclinical findings, the clinical translation of metabolically targeted therapies faces several important challenges. Metabolic pathways are highly conserved between normal and malignant cells, raising concerns about systemic toxicity when key enzymes, such as glycolytic regulators or lipid synthesis enzymes, are inhibited. In addition, tumors often exhibit significant metabolic plasticity, allowing them to compensate for inhibited pathways by activating alternative metabolic routes (134). This adaptability can contribute to therapeutic resistance and may partly explain why some metabolic inhibitors have shown limited efficacy as single agents in clinical trials. Another challenge arises from the complex metabolic interactions within the tumor microenvironment, where targeting tumor metabolism may inadvertently affect immune cell function. For example, excessive inhibition of glycolysis or amino acid metabolism could impair effector T cell activity while simultaneously suppressing tumor growth.

Consequently, many investigators now propose that combination strategies may represent the most effective approach for exploiting tumor immunometabolism therapeutically. Integrating metabolic inhibitors with immune checkpoint blockade, macrophage-reprogramming agents, or anti-angiogenic therapies may simultaneously disrupt tumor metabolic support while restoring immune effector function (135). Such combination approaches may also reduce the likelihood of metabolic escape mechanisms. Continued efforts to identify context-specific metabolic vulnerabilities, along with improved patient stratification based on metabolic biomarkers, will be essential for translating immunometabolic therapies into durable clinical benefit.

## 8 Clinical implications of TAM immunometabolism

Tumor-associated macrophages (TAMs) have emerged as pivotal determinants of clinical outcomes in solid tumors, with their density, phenotype, and spatial localization linked to prognosis, treatment resistance, and patterns of metastatic spread. High infiltration of M2-like TAMs is consistently associated with poor survival, diminished responses to immune checkpoint inhibitors, and increased likelihood of recurrence, underscoring their value as prognostic and potentially predictive biomarkers. Their ability to induce CD8^+^ T-cell exhaustion through PD-L1, VISTA, B7-H4, and metabolic deprivation explains why many tumors remain immunologically “cold” and refractory to current immunotherapies. The growing recognition that tumor-derived metabolites, particularly lactate, fatty acids, and tryptophan derivatives, actively reprogram TAMs toward immunosuppressive states highlights a therapeutic vulnerability. Metabolic inhibitors targeting lactate transport, lipid synthesis, glutamine metabolism, and IDO1/TDO pathways may enhance immunotherapy efficacy by disrupting TAM-driven immune tolerance. These approaches gain further relevance in cancers characterized by metabolic dysregulation, such as breast, pancreatic, and hepatocellular carcinoma.

Clinically, TAM-targeted therapies are being implemented. CSF1R inhibitors have demonstrated activity in select malignancies, while macrophage-reprogramming agents, STAT3 inhibitors, and COX-2 blockade show promise in reshaping the tumor microenvironment to improve treatment response. The emergence of engineered macrophage therapies, such as CAR-macrophages, offers a transformative approach for solid tumors that are traditionally resistant to T-cell therapies. Integrating TAM modulation with existing cancer treatments may represent the next significant advance in oncology. Rational combinations with immune checkpoint inhibitors, anti-angiogenic agents, chemotherapy, and metabolic therapies have the potential to convert immunosuppressive TMEs into therapeutically responsive ecosystems. As our mechanistic understanding deepens, TAM-focused interventions may become essential components of precision oncology and personalized immunotherapy strategies.

### 8.1 Interspecies differences in macrophage immunometabolism and translational implications

While murine models have provided foundational insights into macrophage immunometabolism, significant metabolic and functional differences exist between mouse and human macrophages (3). These interspecies disparities have important implications for the interpretation and translation of preclinical findings. Human macrophages exhibit distinct metabolic preferences compared to their murine counterparts, including differences in nitric oxide production, arginine metabolism, and reliance on glycolysis versus oxidative metabolism (136). For example, inducible nitric oxide synthase (iNOS) activity is robust in murine inflammatory macrophages but is comparatively limited in human macrophages, altering redox balance and antimicrobial mechanisms. Lipid handling and cholesterol metabolism also differ substantially, influencing macrophage polarization and inflammatory potential **Figure 10**.

**Figure 10:**
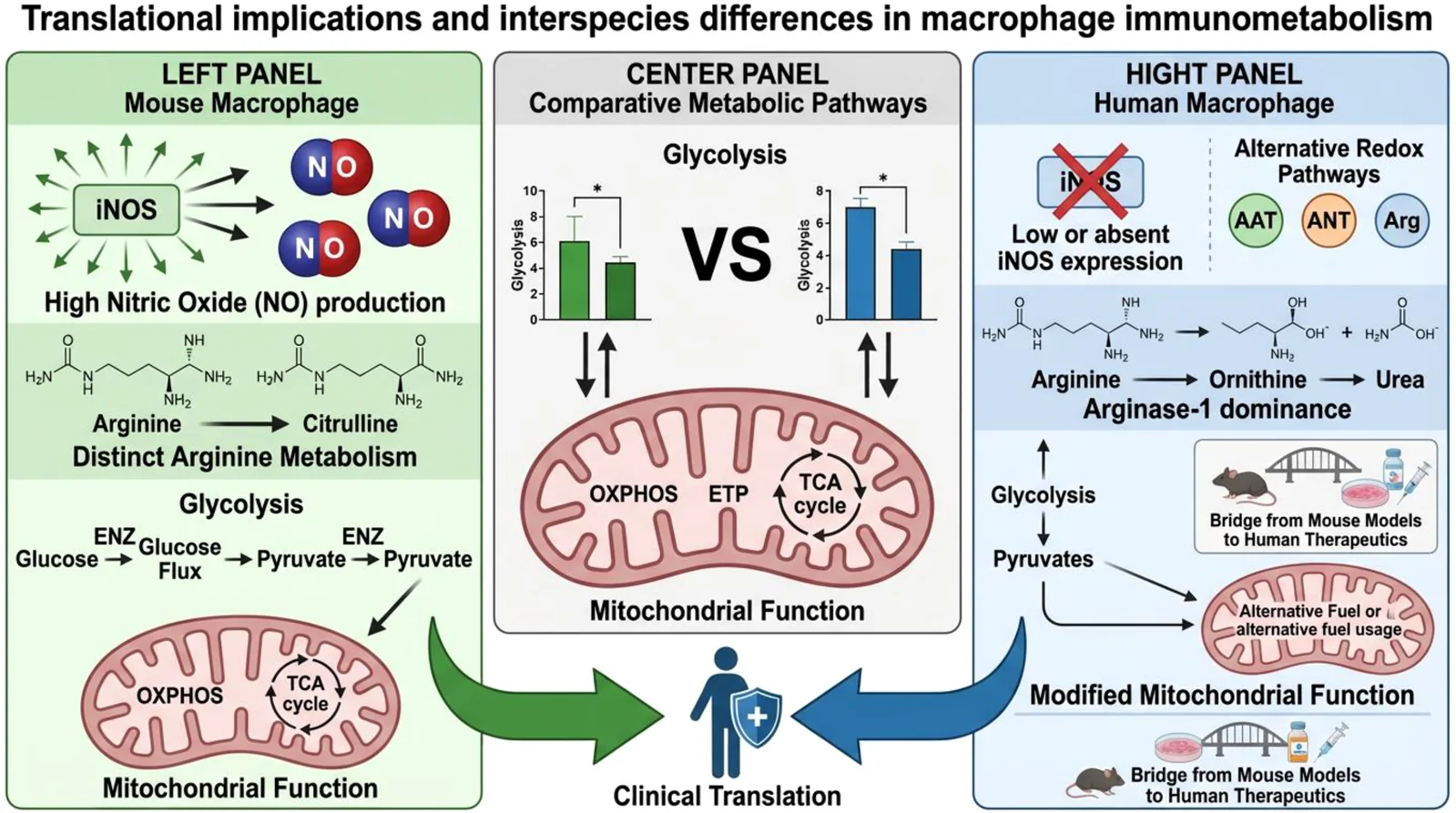
Translational differences between murine and human macrophage metabolism. Important interspecies differences in arginine metabolism, nitric oxide production, and mitochondrial activity influence the translation of immunometabolic findings to human disease.

Single-cell and metabolomic studies further reveal that human macrophages display greater metabolic heterogeneity across tissues, shaped by local nutrient availability and microenvironmental signals (11). These differences underscore the need for caution when extrapolating murine immunometabolic pathways to human disease and highlight the importance of human-based experimental systems. Future immunometabolic therapies targeting macrophages must account for these interspecies variations to ensure translational success. Integrating data from human tissues, organoid systems, and advanced metabolic profiling will be critical for refining macrophage-targeted interventions.

### 8.2 Controversies and divergent perspectives in macrophage immunometabolism

Despite rapid progress in the field, several conceptual debates remain unresolved in macrophage immunometabolism. One major controversy concerns the validity of the classical M1/M2 polarization framework, which is increasingly challenged by single-cell and spatial profiling studies revealing continuous and context-dependent macrophage states rather than discrete phenotypes. Additionally, conflicting evidence exists regarding the therapeutic reprogrammability of tumor-associated macrophages, with some studies suggesting that they can be effectively reprogrammed. In contrast, others indicate that prolonged exposure to the tumor microenvironment imposes irreversible suppressive programs. The role of metabolic interventions in combination with immune checkpoint blockade also remains debated, as metabolic targeting may either enhance or inadvertently dampen antitumor immunity depending on timing and context.

### 8.3 Current knowledge gaps in macrophage immunometabolism

Several critical knowledge gaps limit the translation of macrophage immunometabolism into clinical applications. The causal relationships between specific metabolic pathways and stable macrophage functional states remain incompletely defined, particularly in complex tissue environments where multiple metabolic signals coexist. Furthermore, interspecies differences between murine and human macrophages, especially in nitric oxide biology, lipid handling, and amino acid metabolism, complicate extrapolation from preclinical models. A lack of standardized metabolic profiling approaches and limited longitudinal human data further constrain efforts to identify actionable metabolic checkpoints in disease.

### 8.4 Tumor-associated macrophages as a model system for macrophage immunometabolism

Although this review emphasizes tumor-associated macrophages, the intent is not to restrict immunometabolic insights to oncology alone. Instead, TAMs serve as a well-characterized and experimentally accessible model for understanding how metabolic reprogramming shapes macrophage function under conditions of hypoxia, nutrient competition, and chronic signaling stress. Many of the immunometabolic principles discussed, such as glycolytic bias, lipid-mediated signaling, and amino acid-dependent immune regulation, are also relevant to macrophages in infectious, autoimmune, and metabolic diseases. Thus, conclusions drawn from TAM biology provide a transferable framework for broader macrophage-driven pathologies.

## 9 Conclusion and future perspectives

Macrophage immunometabolism has moved from a descriptive concept to a mechanistic framework that explains how immune phenotypes are established, stabilized, and reshaped by tissue context. In this context, tumor-associated macrophages provide a particularly informative model because their metabolic states are continuously shaped by tumor-derived metabolites, hypoxia, and nutrient competition within the tumor microenvironment. The evidence synthesized in this review supports a central conclusion: macrophage polarization and function are constrained by metabolic circuitry, particularly glucose flux and mitochondrial respiration, lipid uptake and signaling, cholesterol balance, and amino acid–derived immunoregulatory metabolites. Within tumors, TAMs exemplify this principle because the tumor microenvironment imposes hypoxia and nutrient limitation while providing abundant metabolites such as lactate, lipid mediators, and tryptophan- or arginine-derived products that bias macrophages toward immunosuppressive programs. These metabolic shifts amplify checkpoint signaling, impair cytotoxic lymphocyte activity through nutrient deprivation, and promote angiogenesis, invasion, and matrix remodeling, collectively shaping clinical outcomes. An important emerging theme is that immunometabolism also governs macrophage survival and immunological outputs through regulated cell death pathways. Ferroptosis provides a particularly compelling bridge between metabolism and immunity: iron handling, lipid composition, antioxidant capacity, and mitochondrial redox status converge to determine ferroptotic sensitivity, while ferroptotic signals can remodel local inflammation and immune priming. Positioning ferroptosis within the broader immunometabolic landscape opens new opportunities to reprogram macrophages in cancer and beyond.

Future work should prioritize four directions. First, macrophage metabolic states must be interpreted as context-dependent continua rather than fixed M1/M2 categories; integrating single-cell, spatial, and metabolic profiling will be essential to map functional states to nutrient landscapes in tissues. Second, mechanistic studies should resolve causal “metabolic checkpoints” that lock macrophages into suppressive versus protective trajectories, including lactate sensing, lipid droplet biology, cholesterol efflux pathways, and amino acid–linked epigenetic remodeling. Third, therapeutic development should shift from listing individual inhibitors toward strategy-driven combinations that co-target tumor metabolism and TAM programs, with rational pairing to checkpoint blockade, anti-angiogenic agents, and emerging macrophage-engineering approaches such as CAR-macrophages. Fourth, interspecies differences in nitric oxide biology, arginine metabolism, and mitochondrial programs between mouse and human macrophages must be addressed with human tissue datasets and more predictive experimental systems to improve translation.

Overall, bridging metabolic and immune pathways in macrophages provides a unifying explanation for macrophage plasticity and offers actionable therapeutic entry points. As immunometabolic targets mature, macrophage-focused metabolic interventions are likely to become key components of combination immunotherapy and precision approaches for macrophage-driven pathologies.
